# Dietary Micronutrient Management to Treat Mitochondrial Dysfunction in Diet-Induced Obese Mice

**DOI:** 10.3390/ijms22062862

**Published:** 2021-03-11

**Authors:** Fabiano Cimmino, Angela Catapano, Giovanna Trinchese, Gina Cavaliere, Rosanna Culurciello, Chiara Fogliano, Eduardo Penna, Valeria Lucci, Marianna Crispino, Bice Avallone, Elio Pizzo, Maria Pina Mollica

**Affiliations:** 1 Department of Biology, University of Naples Federico II, 80126 Naples, Italy; fabiano.cimmino@unina.it (F.C.); angelacatapano@me.com (A.C.); giovanna.trinchese@unina.it (G.T.); gina.cavaliere@unina.it (G.C.); rosanna.culurciello@unina.it (R.C.); chiara.fogliano@unina.it (C.F.); eduardo.penna@unina.it (E.P.); valeria.lucci@unina.it (V.L.); crispino@unina.it (M.C.); bice.avallone@unina.it (B.A.); eliodoro.pizzo@unina.it (E.P.); 2 Department of Pharmacy, University of Naples Federico II, 80131 Naples, Italy; 3 IEOS, Institute of Experimental Endocrinology and Oncology “G. Salvatore”—National Research Council, 80131 Naples, Italy

**Keywords:** mitochondria, non-communicable diseases, oxidative stress, inflammation, obesity, micronutrients, dimethylglycine, vitamin B

## Abstract

Obesity and associated metabolic disturbances, which have been increasing worldwide in recent years, are the consequences of unhealthy diets and physical inactivity and are the main factors underlying non-communicable diseases (NCD). These diseases are now responsible for about three out of five deaths worldwide, and it has been shown that they depend on mitochondrial dysfunction, systemic inflammation and oxidative stress. It was also demonstrated that several nutritional components modulating these processes are able to influence metabolic homeostasis and, consequently, to prevent or delay the onset of NCD. An interesting combination of nutraceutical substances, named DMG-gold, has been shown to promote metabolic and physical wellness. The aim of this research was to investigate the metabolic, inflammatory and oxidative pathways modulated by DMG-gold in an animal model with diet-induced obesity. Our data indicate that DMG-gold decreases the metabolic efficiency and inflammatory state and acts as an antioxidant and detoxifying agent, modulating mitochondrial functions. Therefore, DMG-gold is a promising candidate in the prevention/treatment of NCD.

## 1. Introduction

Non-communicable diseases (NCD), including obesity, cardiovascular diseases, diabetes, cancers, autoimmune diseases and depression [1,2,3], have a major impact on disability and premature death globally, accounting for up to 72% of world deaths [4]. The development of NCD depends on chronic low-grade inflammation, characterized by high concentrations of circulating proinflammatory cytokines and oxidative stress. An important modulator of chronic inflammation and oxidative stress is nutritional habit [5,6,7,8]. Therefore, it is not surprising that diet is widely accepted as a key determinant of NCD, together with lifestyle [9,10]. Epidemiological studies linking nutrition and diseases have highlighted two different situations responsible for micronutrient deficiency: from one point of view, most of the population in developing countries suffers from insufficient nutrient intake and calories, leading to multiple vitamin deficiencies; on the other hand, in industrialized countries, the so-called “diseases of well-being” are responsible for nutritional imbalances produced by excessive caloric intake, high saturated fat content, low fiber content and reduced intake of vegetables, leading to micronutrient deficiencies and damage from oxidizing substances. These harmful eating habits have facilitated the worldwide usage of micronutrient supplements in the form of pills, tablets, capsules or liquid, called nutraceuticals. They are not considered drugs, but are intended as concentrated sources of nutrients, specifically minerals and vitamins, and a very wide variety of other substances as amino acids, essential fatty acids, fibers and extracts of various plants and herbs [11], having nutritional and metabolic effects. Indeed, they can correct nutritional deficiencies, improving or assisting specific physiological functions. The dietary supplement object of this study is DMG-gold based on dimethylglycine (DMG), trimethylglycine (TMG) and vitamins B1, B2, B3 (Niacin), B6 and B12. In particular, dimethylglycine and group B vitamins act on the regulation of mitochondrial energy metabolism and protect the body from damage caused by free radicals, promoting the reduction in tiredness and fatigue [12].

Dimethylglycine, involved in glycine metabolism, is a source of glycine for glutathione synthesis, thus improving the antioxidant capacity of the body [13,14]. Moreover, dimethylglycine, acting as a methyl donor, functions as an antioxidant to prevent oxidative stress and scavenge excess of free radicals [15].

B vitamins participate in a complex network of reactions involved in the regulation of energy metabolism and therefore are essential for mitochondrial activity. Vitamin B1 (thiamine) is recognized as a cofactor for mitochondrial enzyme complexes that is involved in intermediary metabolism responsible for energy production. Accordingly, thiamine deficiency is associated with disorders in adenosine triphosphate (ATP) production [16] and reduction in fatty acid oxidation [17]. Vitamin B1 is also a cofactor of transketolase, a cytosolic enzyme involved in the pentose phosphate pathway which plays a major role in the production of nicotinamide adenine dinucleotide phosphate-hydrogen (NADPH) for maintaining cellular redox status, glutathione (GSH) levels and protein sulphydryl groups, as well as fatty acid synthesis.

Vitamin B2 (riboflavin) is a precursor of flavin adenine dinucleotide (FAD) and flavin mononucleotide, which are essential for the activity of flavoenzymes including oxidases, reductase and dehydrogenases, and act as electron carriers. Indeed, it is well known that vitamin B2 deficiency induces fatty liver [18] and prevents hepatic lipid peroxidation [19].

Vitamin B3 (niacin) is a precursor of reduced nicotinamide adenine dinucleotide (NAD+) and nicotinamide adenine dinucleotide phosphate (NADP+). These molecules are involved in mitochondrial respiration, glycolysis and lipid β-oxidation. Dietary vitamin B3 intake attenuates dyslipidemia [20] and hepatic steatosis, modulating hepatic lipogenesis [21] and lipid oxidation [22], and oxidative/inflammatory processes [23].

Vitamin B6 plays a key role in mitochondria, acting as a coenzyme for transaminases involved in the catabolism of all amino acids [24]. The protective role of vitamin B6 against hepatic lipid accumulation was shown both in an animal model of diet-induced obesity [25] and in patients affected by nonalcoholic fatty liver disease (NAFLD) [26], indicating the impact of vitamin B6 on the lipid metabolism.

Emerging evidence highlighted that excessive energy intake and, particularly, inadequate fat processing may evoke complex biochemical processes such as inflammation, oxidative stress and impairment of mitochondrial function [27]. The liver plays a central role in the development of obesity-associated metabolic alterations. Indeed, hepatic mitochondrial dysfunction can cause an alteration of fat oxidation, reactive oxygen species (ROS) production and oxidative stress [28]. Therefore, molecules able to modulate mitochondrial function and efficiency are advocated for the prevention/treatment of obesity and related diseases [29].

Thus, it is demonstrated that both dimethylglycine and group B vitamins have independent beneficial effects on metabolism and mitochondrial functions. The aim of our study was to investigate the effects of the combination of these compounds, in the form of DMG-gold, on mitochondrial functions and efficiency. In particular, we analyze how modulation of mitochondrial activity, DMG-gold-dependent, may affect body composition and inflammatory and oxidative states in obese mice fed a high-fat diet (HFD).

## 2. Results

### 2.1. DMG-Gold Supplementation Reduces Lipid Accumulation and Increases Energy Expenditure and Resting Metabolic Rate

**Figure 1:**
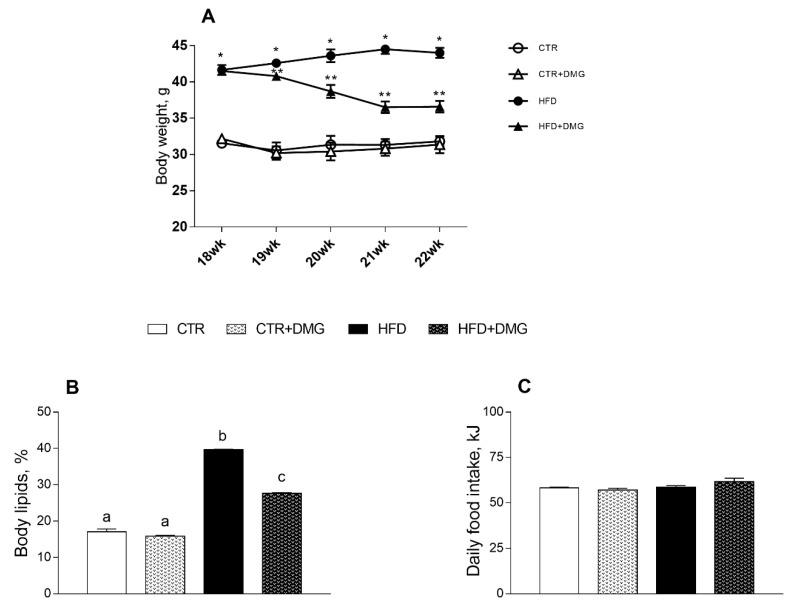
DMG-gold treatment reduces body weight and lipid contents in high-fat diet (HFD)-fed mice. Body weight from 18th to 22nd weeks (**A**) is shown (* *p* < 0.05 vs. CTR; ** *p* < 0.05 vs. HFD). Body lipids, expressed as percentage of body weight (**B**), and daily food intake (**C**) values are reported. Data are presented as means ± SEM from *n* = 7 animals/group. CTR: control; HFD: high-fat diet; DMG: DMG-gold. Different superscripted letters on the histograms (a,b,c) indicate significantly different average values, *p* < 0.05.

Animals treated with an HFD for 22 weeks compared to control group (CTR), fed standard diet, display an increase in body weight and in body lipids content, in agreement with previous data [30]. These increases were partially reversed by the supplementation with DMG-gold during the 4 weeks of supplemented dietary treatment. (Figure 1A,B). All the animal groups had the same food intake (Figure 1C).

**Figure 2:**
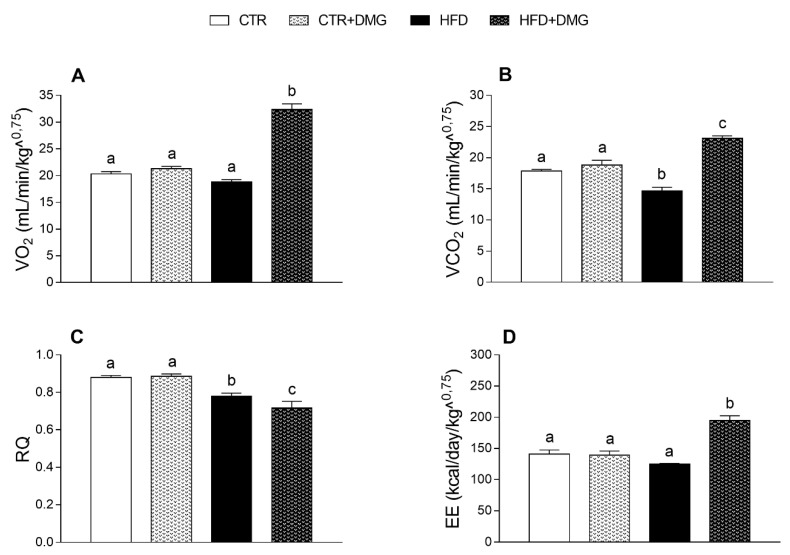
DMG-gold increased energy expenditure in high-fat diet (HFD)-fed animals. Oxygen consumption (VO_2_) (**A**) and carbon dioxide production (VCO_2_) (**B**), respiratory quotient (RQ) (VCO_2_/VO_2_) (**C**) and energy expenditure (EE) (**D**) were determined at the end of the experimental period by an open-circuit calorimeter (*n* = 7 each group). Data are presented as means ± SEM. CTR: control, HFD: high-fat diet, DMG: DMG-gold. Different superscripted letters on the histograms (a,b,c) indicate significantly different average values, *p* < 0.05.

DMG-gold supplementation increased energy metabolism in HFD-treated mice, as shown by the higher oxygen consumption (VO_2_) and carbon dioxide production (VCO_2_) (Figure 2A,B) compared to the other group. Notably, the respiratory quotient (RQ) ratio decreased in DMG-gold-treated HFD mice compared to HFD animals, indicating an increase in fatty acid oxidation (Figure 2C). Consistently, the energy expenditure increased in the HFD mice supplemented with DMG-gold compared to the other group (Figure 2D). The VCO_2_ production and RQ ratio significantly decreased in HFD mice compared to the control (Figure 2B,C).

### 2.2. DMG-Gold Supplementation Modulates Serum and Hepatic Inflammatory Markers and Metabolic Parameters

**Figure 3:**
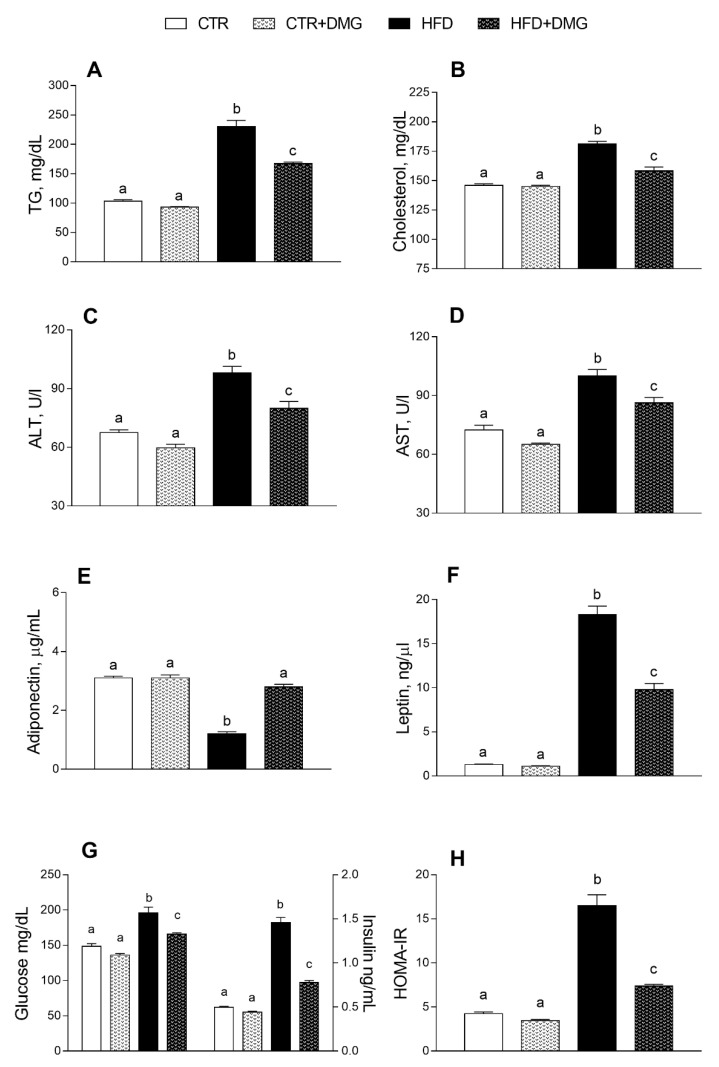
DMG-gold restored serum metabolic parameters and hepatic markers in serum of HFD animals. Metabolic parameters, such as triglycerides (**A**) and cholesterol (**B**), alanine aminotransferase (ALT) (**C**), aspartate aminotransferase (AST) (**D**), adiponectin (**E**), leptin (**F**), glucose and insulin (**G**) and insulin resistance-homoeostatic model assessment (IR-HOMA) index (**H**), were measured in serum. Data are presented as means ± SEM from *n* = 7 animals/group. CTR: control, HFD: high-fat diet, DMG: DMG-gold. Different superscripted letters on the histograms (a,b,c) indicate significantly different average values, *p* < 0.05.

**Figure 4:**
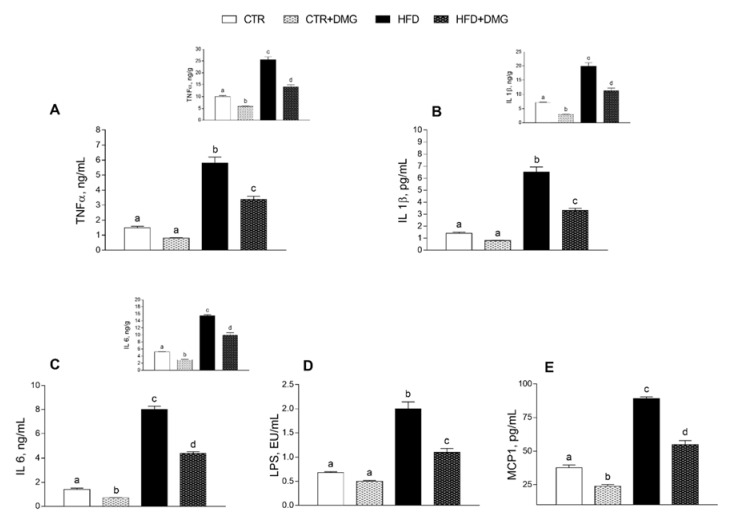
DMG-gold supplementation reduced inflammatory parameters in the liver and in the serum in both HFD and control animals. Proinflammatory cytokines, such as tumor necrosis factor (TNF-α) (**A**), interleukin-1β (IL-1β) (**B**), interleukin-6 (IL-6) (**C**), lipopolysaccharide (LPS) (**D**) and monocyte chemoattractant protein-1 (MCP-1) (**E**), were measured in the serum and in the liver (upper panels). Data are presented as means ± SEM from *n* = 7 animals/group. CTR: control, HFD: high-fat diet, DMG: DMG-gold. Different superscripted letters on the histograms (a,b,c,d) indicate significantly different average values, *p* < 0.05.

Serum triglycerides, cholesterol, alanine aminotransferase (ALT) and aspartate aminotransferase (AST) were significantly increased in HFD compared to control mice (Figure 3A–D). Interestingly, this increase was partially reversed by DMG-gold supplementation to HFD animals (Figure 3A–D). Similarly, the HFD-dependent alteration of serum levels of adiponectin and leptin was partially reversed by DMG-gold treatments (Figure 3E,F). As expected, HFD intake increased glycaemia and insulin levels, but these levels were significantly reduced by DMG-gold supplementation (Figure 3G). Similarly, the HFD-DMG-gold-treated group exhibited a marked reduction in the insulin resistance-homoeostatic model assessment (IR-HOMA) index compared to the HFD group (Figure 3H). The proinflammatory serum markers increased by the HFD, such as tumor necrosis factor-α (TNF-α), interleukin 1-β (IL-1-β), interleukin-6 (IL-6), lipopolysaccharide (LPS) and monocyte chemoattractant protein-1 (MCP-1), were also significantly reduced in DMG-gold-supplemented HFD animals (Figure 4). Moreover, DMG-gold also reduced these inflammatory parameters in the control group, although the decrease was significant only for IL-6 and MCP-1 (Figure 4). The anti-inflammatory effect of DMG-gold in control and HFD mice was also confirmed in the hepatic tissue (insets of Figure 4A–C).

### 2.3. DMG-Gold Supplementation Decreases Hepatic Lipid Depots

**Figure 5:**
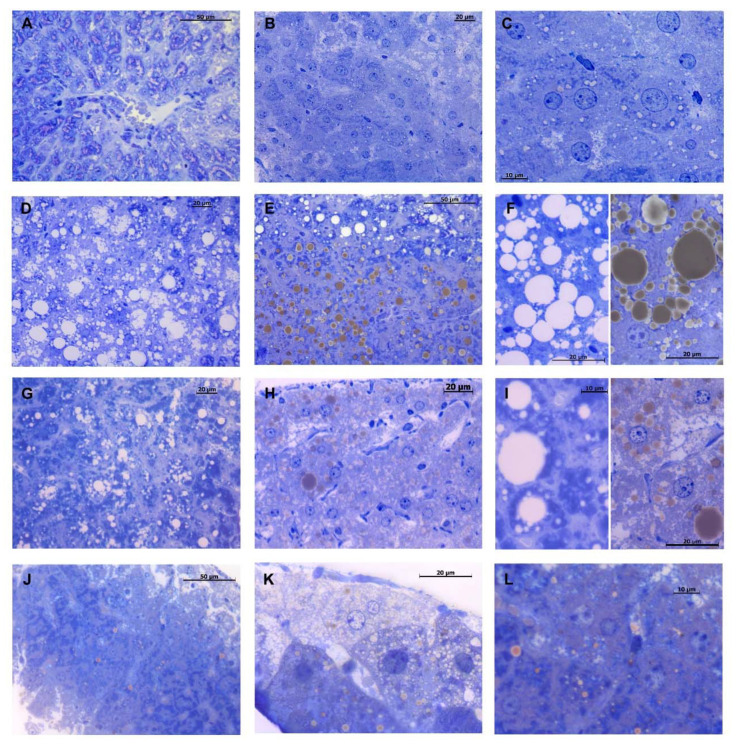
Toluidine blue staining of liver semithin sections. Control group shows a regular hepatic morphology with few little drops of unsaturated lipids (brown-colored) visible within the hepatocyte’s cytoplasm (**A**–**C**). HFD mice show a noticeable hepatosteatosis condition, characterized by hepatocytes with a wide intracytoplasmic spread of both saturated (white-colored) and unsaturated lipid drops of various sizes (**D**–**F**). DMG-treated mice fed with HFD show improvement in steatosis, reduction in fat deposits and substantial decrease in lipid drop size (**G**–**I**). DMG-treated mice show a slight increase in the size of lipid drops, compared to the control group (**J**–**L**). Magnification 40× in (**A**,**B**,**D**,**E**,**G**,**H**,**J**,**K**); 100× in (**C**,**F**,**I**,**L**).

In the control group, light microscopy analysis of hepatic tissue showed a normal hepatic architecture with polyhedral cells, radiating from the centrilobular vein, with a centrally located nucleus (Figure 5A). Sinusoids lined with endothelial cells, and bile canaliculi were visible among hepatocytes. Moreover, it was possible to observe a phenomenon of metachromasia given by the presence of glycogen storage within the hepatocytes (Figure 5A). A slight quantity of saturated fat was observed inside the hepatocyte’s cytoplasm, with small-size lipid droplets accumulated mainly around the centrilobular vein but faintly visible or totally lacking in the peripheral area (Figure 5B,C). Unsaturated fat was almost completely absent. Conversely, HFD mice showed severe hepatic steatosis around the liver central area but also towards the periphery, revealed by mixed macro- and micro-drops of intracytoplasmic fat accumulation, made by both saturated and unsaturated fats (Figure 5D,E). Hepatocytes showed an altered morphology with a peripheral location of the nucleus due to the large accumulation of variable-sized lipid drops (Figure 5F). Furthermore, neither bile canaliculi nor the sinusoids were visible, as they were hidden by lipid drops in the most steatotic areas. For the same reason, metachromasia given by glycogen was also missing. Interestingly, DMG treatment leads to an improvement in hepatic steatosis of HFD mice. Indeed, in DMG-treated HFD mice, a decrease in fat content, especially saturated fat, was observed (Figure 5G,H). Although the hepatic architecture was not fully re-established, hepatocytes regained the normal morphology, with the lipid drops mainly located around the liver central area and a lower percentage in the peripheral area (Figure 5I). A slight increase in fat, predominantly in the amount and in the size of the unsaturated lipid droplets, was detected in DMG-treated mice fed with a standard diet (Figure 5J,K). Nonetheless, lipid drops were found towards the central area rather than in the periphery zone and the hepatocytes were well delineated, displaying a normal morphology (Figure 5L). The cytological investigations show that hepatic fibrosis is not noticeable in all experimental groups (data not shown).

### 2.4. Modulation of Hepatic Mitochondrial Efficiency and Oxidative Stress by DMG-Gold Treatment

**Figure 6:**
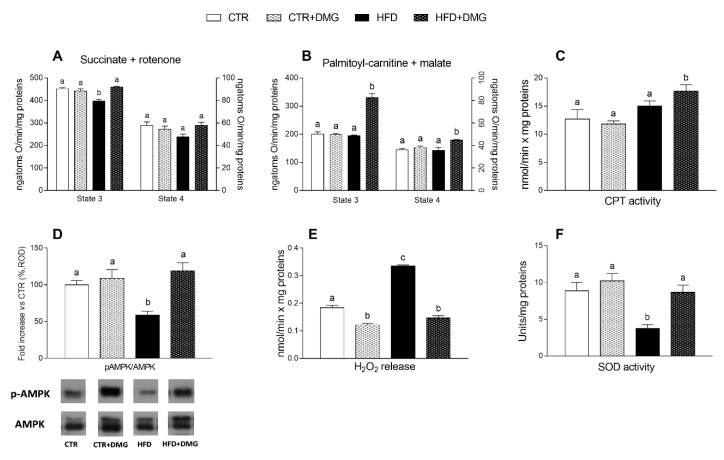
Effect of DMG-gold on liver mitochondrial function. Mitochondrial respiration in the presence of succinate and rotenone (**A**) or palmitoyl-carnitine and malate (**B**) as substrates was determined in the presence (state 3) or absence (state 4) of adenosine diphosphate (ADP). (**C**) Carnitine palmitoyl transferase (CPT) activity in mitochondria is shown. (**D**) Ratio of pAMPK/AMPK from total liver extract of all animal groups, with representative immunoblots. The values of treated groups are expressed as percentage of the CTR group. (**E**) Hydrogen peroxide (H_2_O_2_) release and (**F**) superoxide dismutase (SOD) activity were determined in hepatic isolated mitochondria. Data are presented as means ± SEM from *n* = 7 animals/group, except for panel D (*n* = 5). CTR: control, HFD: high-fat diet, DMG: DMG-gold. Different superscripted letters on the histograms (a,b,c) indicate significantly different average values, *p* < 0.05.

The mitochondrial state 3 respiratory rate, evaluated using succinate and rotenone as substrates in the presence of adenosine diphosphate (ADP), was decreased in HFD-fed animals compared with the other groups and restored by DMG-gold (Figure 6A). To evaluate fatty acid oxidation, the state 3 respiratory rate was measured using palmitoyl-carnitine and malate as substrates; in HFD-fed animals, DMG-gold administration increased the oxygen consumption rate compared with standard diet- and HFD-fed groups (Figure 6B). No variation was observed in the mitochondrial state 4 respiratory rate among all groups using both succinate and palmitoyl-carnitine as substrates (Figure 6A,B). No difference in carnitine palmitoyl transferase (CPT) activity was observed between standard diet- and HFD-fed mice, while a significant increase was shown in DMG-treated groups (Figure 6C). We evaluated adenosine monophosphate-activated protein kinase (AMPK) expression and phosphorylation levels in total liver extract, by Western blot analysis. AMPK activation was significantly reduced in the liver of HFD groups, but the treatment of these animals with DMG was able to rescue the activation (Figure 6D). The yield of hydrogen peroxide (H_2_O_2_) increased in HFD compared to control animals. Interestingly, DMG treatment significantly reduced the H_2_O_2_ levels in both control and HFD mice (Figure 6E). Superoxide dismutase (SOD) activity was significantly lower in HFD mice compared to control mice, but it was restored to the control level in HFD animals supplemented with DMG-gold (Figure 6F). Altogether, these data suggest that the administration of DMG-gold results in reduced mitochondrial ROS production.

### 2.5. DMG-Gold Supplementation Increases Antioxidant/Detoxifying Defenses

**Figure 7:**
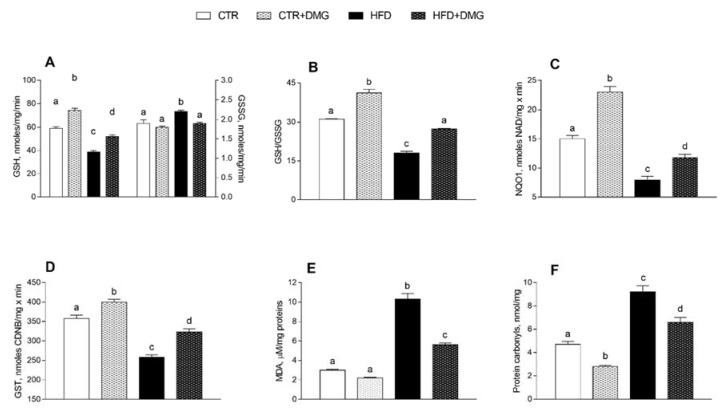
DMG-gold improved antioxidant/detoxifying defenses and reduce peroxidation products. Reduced glutathione (GSH) and oxidized glutathione (GSSG) content (**A**), GSH-to-GSSG ratio (**B**), NAD(P)H quinone dehydrogenase (NQO1) (**C**) and glutathione transferase (GST) (**D**) activities and malondialdehyde (MDA) (**E**) and protein carbonyl levels (**F**) are shown. Data are presented as means ± SEM from *n* = 7 animals/group. CTR: control, HFD: high-fat diet, DMG: DMG-gold. Different superscripted letters on the histograms (a,b,c,d) indicate significantly different average values, *p* < 0.05.

DMG-gold administration improved the antioxidant state and cytoprotective enzyme activities. Indeed, GSH levels increased significantly in control and HFD animals supplemented with DMG-gold, compared to the corresponding group that did not receive supplementation (Figure 7A). On the other hand, oxidized glutathione (GSSG) levels increased in the HFD group and returned to the control level with DMG-gold supplementation (Figure 7A). Therefore, the GSH/GSSG ratio increased in both control and HFD animals supplemented with DMG-gold (Figure 7B). The activities of NAD(P)H quinone dehydrogenase (NQO1) and glutathione transferase (GST) had the same trend as the GSH/GSSG ratio (Figure 7C,D). In addition, malondialdehyde (MDA) levels increased in HFD mice, but this effect was reduced in animals supplemented with DMG-gold (Figure 7E), and also protein carbonyl (PC) concentrations were reduced by DMG supplementation in both differently fed experimental groups (Figure 7F).

## 3. Discussion

As expected, HFD treatment in mice induced an increase in body weight and lipid gain, metabolic alterations such as dyslipidemia and an increase in low-grade inflammation compared to the standard diet-fed group [31]. Here, we demonstrated that dietary supplementation with DMG-gold induced a significant reduction in body weight in HFD-fed animals and in body lipid levels in differently fed experimental groups, showing a significant impact on the modulation of adiponectin and leptin levels. In HFD-fed animals, the effects of DMG-gold on body weight and lipids can be explained, at least in part, by an increase in energy expenditure and O_2_ consumption and a decreased RQ value that is an index of the ratio of carbohydrate/fatty acid oxidation. These results suggest that HFD mice supplemented with DMG-gold preferentially used fatty acids as metabolic fuel, dissipating the large part of the higher energy intake through increased metabolic activity. Accordingly, DMG-gold attenuated an HFD-induced alteration of cholesterol, triglycerides and hormonal profiles, restoring glycemia and insulinemia levels and reducing inflammation. Moreover, DMG-gold supplementation in HFD mice counteracted adiponectin and leptin alterations. These two adipokines are involved in glucose and lipid metabolism, through AMPK activation [32,33]. AMPK is an important metabolic regulator that controls the mitochondrial fatty acid oxidation, and it is a potent counter-regulator of inflammatory pathways [34]. Our data indicate an increased activity of AMPK in animals of HFD groups supplemented with DMG-gold. Accordingly, DMG-gold ameliorated the alterations of the proinflammatory markers induced by the HFD. In addition, DMG-gold supplementation reduced, in both standard diet- and HFD-fed groups, the serum level of IL-6 and hepatic inflammatory parameters. Moreover, in HFD-fed animals, DMG-gold supplementation lowered the levels of ALT and AST, two toxicity markers that increased in HFD animals as a consequence of the hepatic injury caused by the overload of lipid intake [35].

Two of the consequences of oxidative stress in HFD animals are increased lipid peroxidation (measured by MDA levels) and protein carbonyls (CP), which physiologically occur at lower levels in the organism [36,37], but they increased in HFD animals as a consequence of oxidative stress. Excess of ROS in cells, associated with an impaired enzymatic antioxidant activity, can lead to cell and tissue damage. Since the main sites of ROS production are mitochondria, we analyzed the modulation of liver mitochondrial function following the administration of DMG-gold. Our data confirm the association between HFD-induced ectopic fat storage in the liver and alterations in the mitochondrial compartment [38]. Indeed, liver mitochondria from the HFD mice exhibited reduced mitochondrial respiratory capacity and increased oxidative stress. We observed that the treatment with DMG-gold in HFD-fed mice is able to increase the mitochondrial respiratory capacity and fatty acid oxidation. This increase in fatty acid oxidation is associated with an enhancement of CPT activity, which would further increase entry of long-chain free fatty acids (FFAs) into mitochondria. The increased lipid oxidation may explain the decreased content of lipids in the liver and the diminished dyslipidemia levels exhibited by these mice. The enhancement of CPT activity may depend on AMPK modulation. Indeed, the activation of AMPK decreases the expression of lipogenic genes [39] and increases the phosphorylation of acetyl-CoA carboxylase (ACC), leading to a reduction in malonyl-CoA, which regulates fatty acid oxidation through the inhibition of CPT [40]. Moreover, the AMPK signaling pathway is the main target of adiponectin in promoting fatty acid oxidation [27], and we observed that the levels of adiponectin were recovered with DMG treatment in HFD-fed animals, strengthening the key role of AMPK activation in the regulation of metabolic and inflammatory cellular homeostasis. Our results suggest the possible influence of AMPK on mitochondrial function, in agreement with a recent report indicating the role of AMPK as a central integrator of mitochondrial homeostasis [41].

The increased fatty acid oxidation observed in HFD-DMG-gold animals may explain the decreased lipid content observed in their liver. In HFD mice, the steatotic condition leads to an inflammatory state, which is reflected in an altered liver architecture. In fact, because of the wide fat storage, hepatocytes appear to be abnormal, and bile canaliculi and sinusoids are not visible. DMG-gold treatment provides an improvement in the hepatic steatosis condition of obese mice livers, with a regain of hepatocytes’ normal morphology and an overall reduction in lipid deposits. In DMG-treated animals’ livers, a decrease in the total amount of saturated fat was detected, in comparison with HFD mice. As a consequence of the reduction in the steatotic condition, a reduction in the inflammatory state was visible, with an improvement in the hepatic architecture. The decrease in fats, mainly saturated, found in DMG-gold HFD mice is in agreement with a decrease in total cholesterol detected by enzymatic analyses. The beneficial effects of DMG-gold on the hepatic lipid metabolism could be attributable, at least in part, to the well-known properties of its main components. B vitamins are involved in the regulation of cell energy metabolism, with a special focus on the lipid metabolism [42]. In particular, previous studies both in an animal model and in humans suggested that higher niacin consumption may be used as a therapeutic tool for improving hepatic steatosis [23]. On the other hand, it is noteworthy that older studies demonstrated that excess niacin induced fatty liver in albino rats fed a 40% HFD for 42 days [43]. More recently, it was demonstrated that a high dose of niacin administrated for 15 weeks increased hepatic steatosis in B6129 mice fed with an HFD, whereas it had no impact on C57BL/6J mice [44].

Moreover, according to the reduced oxidative stress after DMG treatment, we observed reduced levels of MDA, an index of lipid peroxidation, that are particularly relevant since lipid peroxidation is the first step on the way to the development of insulin resistance and its associated diseases [45]. The obtained results are in line with compelling evidence demonstrating the significant antioxidant potential of the micronutrient components of DMG-gold. In particular, dimethylglycine, the main component of DMG-gold, acting as a methyl donor, functions as an antioxidant to prevent oxidative stress [46]. In addition, it has been shown that dimethylglycine sodium salt provides protection against lipopolysaccharide-induced oxidative stress in mice, indicating its potent antioxidant effect due to both its radical scavenging activity and its ability to enhance the endogenous antioxidant defense system [47]. Moreover, niacin displayed a protective role against lipid peroxidation [48], while riboflavin has antioxidant effects [19].

The beneficial effects of DMG-gold administration are also indicated by the increased activities of detoxifying enzymes (GST, NQO1), the improvement in redox status (GSH/GSSG ratio), together with the reduction in oxidative stress, as shown by a decrease in H_2_O_2_ production, and an increase in SOD activity. Accordingly, it was reported that mitochondrial SOD upregulation leads to the activation of AMPK [49].

**Figure 8:**
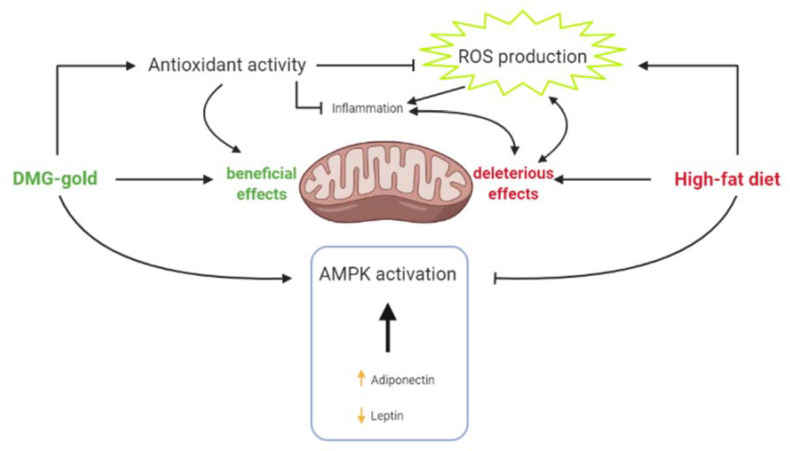
Dietary supplementation with DMG-gold decreases inflammatory and oxidative states, increases detoxifying enzymes’ activity and improves mitochondrial functions and lipid oxidation through AMPK activation in hepatic tissue.

In conclusion, our study highlights that dietary supplementation with DMG-gold decreases inflammatory and oxidative states, increases detoxifying enzymes’ activity and improves mitochondrial functions and lipid oxidation through AMPK activation in hepatic tissue (Figure 8). The observed results allow us to hypothesize that dimethylglycine and B vitamins are the key components responsible for the effects of DMG-gold administration. In particular, the presence of both components in this formulation might potentiate the beneficial effects of each of them on hepatic tissue. These protective effects are particularly relevant in view of the crucial role played by the liver in metabolic homeostasis of the whole body.

## 4. Materials and Methods

### 4.1. Reagents

All chemicals were purchased from Sigma–Aldrich (St. Louis, MO, USA), unless otherwise specified. DMG-GOLD, marketed by the company Erbenobili s.r.l. (Corato, Bari, Italy), is mainly composed of: dymethilglicine (66 mg/mL); inositol (60 mg/mL); betain (TMG, 18 mg/mL); niacin (2.67 mg/mL); vitamin B6 (0.23 mg/mL); vitamin B2 (0.23 mg/mL); vitamin B1 (0.18 mg/mL); vitamin B12 (0.42 mcg/mL), and it was provided in liquid form. The daily administration dose of DMG-GOLD was 450 µL/kg body weight.

### 4.2. Ethics Statement

All procedures involving the animals were carried out in accordance with the international and national law and policies (EU Directive 2010/63/EU for animal experiments, ARRIVE guidelines and the Basel declaration, including the “3R” concept). All animal procedures reported herein were approved by the Institutional Animal Care and Use Committee (CSV) of the University of Naples Federico II under protocol no. 982/2017-PR.

### 4.3. Animals

**Figure 9:**
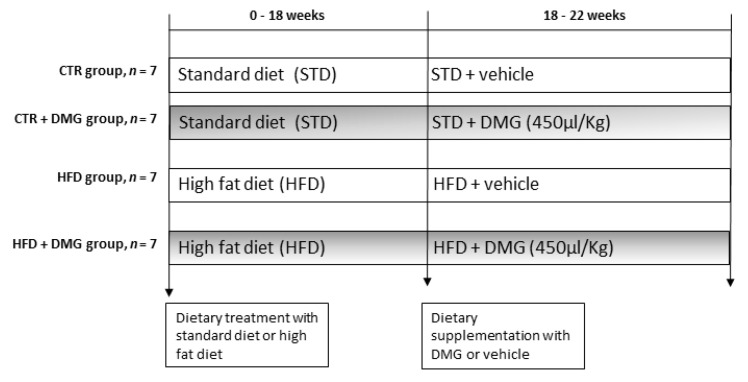
Schematic overview of the experimental design. Male C57Bl/6J (B6) mice (6 weeks old) (*n* = 7 per group) were fed with the standard (STD) or high-fat diet (HFD) for 18 weeks. Then, both STD and HFD mice were treated by gavage with DMG-gold (450 µL/kg/die) for 4 weeks.

Male C57Bl/6J (B6) mice (6 weeks old), purchased from Envigo Srl (San Pietro al Natisone, Udine, Italy), were individually caged in a temperature-controlled room and exposed to a daily 12–12 h light–dark cycle with free access to diet and drinking water. Mice were divided into two experimental groups according to a different dietary regimen: the first group was fed with a standard rodent diet (STD, *n* = 7) (15.88 kJ gross energy/g: 60.4% carbohydrates, 29% protein, 10.6% fat; Mucedola, Milan, Italy); the second group was fed with a high-fat diet (D12451; Research Diets Inc., New Brunswick, NJ, USA) (HFD, *n* = 7) (22.1 kJ/g) in which 40% of metabolizable energy was obtained from lard, and the remaining calories were starch (31%) and protein (29%) (Table 1). After 18 weeks, both STD and HFD mice were divided into two subgroups treated by gavage with the DMG-gold preparation (using 450 µL of /kg/die, as indicated in Section 4.1.) for 4 weeks (CTR+DMG and HFD+DMG, respectively) (Figure 9). This dose was chosen based on the reported daily intake in humans converted to mice [50]. The animals fed with only the standard diet or HFD were orally treated with water as vehicle. Before the sacrifice, the mice were anesthetized by an intra-peritoneal injection of chloral hydrate (40 mg/100 g body weight), and blood was taken via the inferior cava vein and collected in heparin- or EDTA-containing tubes. Livers were removed, aliquots not immediately used for mitochondrial extraction, frozen and stored at −80 °C for further determinations.

During the experimental period, body weight and food intake were monitored daily to calculate weight gain and gross energy intake. Spilled food was collected daily for precise food intake. The gross energy densities for the standard diet and high-fat diet (15.8 and 22.1 kJ/g, respectively) were determined by bomb calorimetry (Parr adiabatic calorimetric, Parr Instrument Co., Moline, IL, USA). Lipid contents in animal carcasses were measured according to Folch [51].

### 4.4. Measurement of Oxygen Consumption (VO_2_), Carbon Dioxide Production (VCO_2_) and Respiratory Quotient (RQ)

Upon an adaption period to the experimental environment, VO_2_ and VCO_2_ were recorded by a monitoring system (Panlab s.r.l., Cornella, Barcelona, Spain) composed of a four-chambered indirect open-circuit calorimeter, designed for continuous and simultaneous monitoring. VO_2_ and VCO_2_ were measured every 15 min (for 3 min) in each chamber for a total of 6 h (from 8:00 a.m. to 14:00 p.m., during the light phase). The mean VO_2_, VCO_2_ and RQ values were calculated by the “Metabolism H” software (Metaox, Metabolism V2.1) [52].

### 4.5. Evaluation of Markers in Blood and in Tissue

Blood samples were centrifuged at 1000× *g* for 10 min at 4 °C. Plasma was removed and stored at −20 °C. Plasma concentrations of triglycerides (TG), cholesterol, alanine aminotransferase (ALT) and aspartate transaminase (AST) were measured by the colorimetric enzymatic method using commercial kits (SGM Italia, Roma, Italy and Randox Laboratories ltd., Antrim, UK). Glucose levels were determined by glucometer (Contour next, Ascensia, Switzerland). Basal fasting values of serum glucose and insulin were used to calculate the homoeostatic model assessment (HOMA) index as (glucose (mg/dL) × insulin (mU/L))/405 [53]. Commercially available ELISA kits were used to determine serum and tissue interleukin-1β (IL-1β), interleukin-6 (IL-6), TNF-α (Thermo Scientific, Rockford, IL, USA; Biovendor R and D, Brno, Czech Republic), adiponectin and leptin (B-Bridge International, Mountain View, CA, USA) and monocyte chemoattractant protein-1 (MCP-1) (Biovendor R&D, Brno, Czech Republic). Lipopolysaccharide (LPS) was measured using the Limulus amebocyte lysate (LAL QCL-1000; Lonza Group Ltd., Basel, Switzerland) technique.

### 4.6. Hepatic Histological Analyses

For each animal (*n* = 4) of all analyzed groups, the liver was cut into 8 blocks of 1 mm^3^, belonging to different areas. Each block was fixed in 2.5% glutaraldehyde + 2.5% paraformaldehyde in 0.1 M PBS pH 7.4, for 4 h at 4 °C, then post-fixed in 1% osmium tetroxide (2 h, 4 °C). After wash series in 0.1 M PBS pH 7.4 at 4 °C, samples were dehydrated in an ascending sequence of ethyl alcohol and propylene oxide and then embedded in Epon 812 (55 °C, 48 h). Semi-thin sections (1.5 µM) were cut with a glass knife for light microscopic observations and stained with 1% toluidine blue solution prepared in 1% sodium tetraborate buffer. Sections obtained were observed with a ZEISS Axiocam microscope camera applied to a Zeiss Axioskop microscope. For each liver area, an average of 30 serial sections were examined to visually quantify the lipid content and the accumulation of saturated fats (white-colored) vs. unsaturated fats (brown-colored), following a previously reported method [54].

### 4.7. Mitochondria Preparation and Analysis

Liver aliquots were finely minced and washed in a medium containing 100 mM KCl, 50 mM Tris-HCl, pH 7.5, 5 mM MgCl_2_, 1 mM EDTA, 5 mM EGTA and 0.1% (*w*/*v*) fatty acid-free bovine serum albumin (BSA). Tissue fragments were homogenized with the above medium (1:8, *w*/*v*) in a Potter Elvehjem homogenizer (Heidolph, Kelheim, Germany) set at 500 rpm (4 strokes = min) and filtered through a sterile gauze. The homogenate was then centrifuged at 1000× *g* for 10 min, and the resulting supernatant was again centrifuged at 3000× *g* for 10 min. The mitochondrial pellet was washed twice and finally resuspended in a medium containing 80 mM LiCl, 50 mM HEPES, 5 mM Tris-PO_4_, 1 mM EGTA and 0.1% (*w*/*v*) fatty acid-free BSA, pH 7.0 [55]. The protein content of the mitochondrial suspension was determined by the method of Hartree [56] using BSA as the protein standard. Mitochondrial oxygen consumption was polarographically measured by a Clark-type electrode (Yellow Springs Instruments, Yellow Springs, OH, USA) at 30 °C. In detail, isolated mitochondria (0.5 mg protein/mL) were incubated in a medium containing 30 mM KCl, 6 mM MgCl_2_, 75 mM sucrose, 1 mM EDTA, 20 mM KH_2_PO_4_ pH 7.0 and 0.1% (*w*/*v*) fatty acid-free BSA. In the presence of 10 mM succinate, 3.75 μM rotenone and 0.6 mM ADP, state 3 oxygen consumption was measured. State 4 was obtained in the absence of ADP. The rate of mitochondrial fatty acid oxidation was assessed in the presence of malate (2.5 mM), palmitoyl-L-carnitine (40 µM) and ADP (0.6 mM). High quality of mitochondrial preparations was indicated by high respiratory control ratio values in all groups (data not shown), calculated as the ratio between states 3 and 4, according to Estabrook [57]. In addition, in control experiments, we assured the quality of our mitochondrial preparation by checking that contamination of mitochondria by other ATPase-containing membranes was lower than 10%, and addition of cytochrome c (3 nmol/mg protein) only enhanced the state 3 respiratory rate by approximately 10% [58].

Carnitine-palmitoyl-transferase (CPT) activity was followed spectrophotometrically as CoA-sH production by the use of 5,5’-dithiobis (nitrobenzoic acid) (DTNB) and, as substrate, palmitoyl-Coa 10 μM. The medium consisted of 50 mM KCl, 10 mM Hepes (pH 7.4), 0.025% Triton X-100, 0.3 mM DTNB and 10–100 pg of mitochondrial protein in a final volume of 1.0 mL. The reaction was followed at 412 nm at 25 °C in a thermostated spectrophotometer, and enzyme activity was calculated from an E412 = 13,600/(M × cm) [59]. Rate of mitochondrial H_2_O_2_ release was assayed by following the linear increase in fluorescence (excitation 312 nm and emission 420 nm) due to the oxidation of homovanillic acid in the presence of horseradish peroxidase [60]. SOD specific activity was measured in a medium containing 0.1 mm EDTA, 2 mm KCN, 50 mm KH_2_PO_4_, pH 7.8, 20 mm cytochrome c, 5 mm xanthine and 0.01 U of xanthine oxidase. Enzyme activity was measured spectrophotometrically (550 nm) at 25 °C, by monitoring the decrease in the reduction rate of cytochrome c by superoxide radicals, generated by the xanthine–xanthine oxidase system. One unit of SOD activity is defined as the concentration of enzyme that inhibits cytochrome c reduction by 50% in the presence of xanthine and xanthine oxidase [61].

### 4.8. Western Blot Analysis

Western blot analysis was performed as previously reported [62]. Briefly, the liver was homogenized at 4 °C in RIPA buffer (50 mM Tris-HCl pH 8, 150 mM NaCl, 1 mM EDTA, 0.1% SDS, 1% Triton-X100, 1× protease inhibitor) and total protein lysates, obtained by centrifugation at 14,000× *g* for 15 min at 4 °C, were subjected to SDS-PAGE and transferred onto a PVDF membrane (IPVH00010 Millipore) using a Bio-Rad Transblot (Bio-Rad, Milan, Italy). Membranes were blocked at room temperature in BSA (3% *w/v* bovine serum albumin, 0.3% *v/v* Tween-20, in PBS) and probed with anti-phospho-AMPKα (1:1000) and AMPK antibodies (1:1000) (Cell Signaling, Danvers, MA, USA). The images were acquired using a Biorad Gel Doc XR System.

### 4.9. Hepatic Lipid Peroxidation, Inflammatory Markers and Antioxidant/Detoxifying Defenses

Lipid peroxidation was determined in liver homogenates by measuring the levels of malondialdehyde (MDA) using the thiobarbituric acid (TBAR) method. The content of malondialdehyde (MDA) was estimated using a colorimetric assay kit as per the manufacturer’s protocol (MAK085, Sigma-Aldrich, St. Louis, MO, USA). Equal volumes (100 μL) of sodium dodecyl sulfate and sample were mixed in a 5 mL conical vial. The mixture was added to 0.4 mL of 1% thiobarbituric acid in 0.2 mL (20%) H_3_PO_4_ and 50 mm NaOH and was mixed gently. The mixture was incubated on ice before being heated for 15 min at 100 °C. The samples were then centrifuged for 10 min at 1600× *g*. Finally, the absorbance of the samples was read at 540 nm. MDA values were expressed as micromoles per milligram of protein [63]. The levels of TNF-α, IL-1b, IL-6 and MCP-1 in liver homogenates were measured using commercially available ELISA kits. In liver homogenates, reduced (GSH) and oxidized (GSSG) glutathione concentrations were measured with the dithionitrobenzoic acid (DTNB)-GSSG reductase recycling assay [64,65], and the GSH/GSSG ratio was used as an oxidative stress marker. To investigate the possible involvement of NF-E2-related factor 2 (Nrf2) in the diet-induced stress, cytoplasmic extracts were prepared from the liver. The enzymatic activities of glutathione S-transferases (GSTs) and quinone-oxidoreductase (NQO1) were evaluated spectrophotometrically in cytoplasmic extracts [66,67,68]. Carbonylated protein accumulation was measured according to Levine [68].

### 4.10. Statistical Analyses

All data were presented as means ± SEM. Differences among groups were compared by one-way ANOVA followed by the Bonferroni post hoc test to correct for multiple comparisons. Differences were considered statistically significant at *p* < 0.05. All analyses were performed using GraphPad Prism (GraphPad Software, San Diego, CA, USA).

## Figures and Tables

**Table 1:** Fatty acid profile of standard rodent diet (STD) (Mucedola s.r.l., Milan, Italy) and HFD (Research Diets Inc., New Brunswick, NJ, USA).

Fatty Acids	STD	HFD
	**%/Total Fat**	**%/Total Fat**
**C10, Capric**	-	0.056
**C12, Lauric**	0.232	0.085
**C14, Myristic**	1.16	1.101
**C15**	-	0.073
**C16, Palmitic**	14.83	19.29
**C16:1, Palmitoleic**	0.929	1.311
**C17**	-	0.353
**C18, Stearic**	3.252	10.38
**C18:1, Oleic**	19.783	33.61
**C18:2, Linoleic**	50.25	29.47
**C18:3, Linolenic**	9.562	2.202
**C20, Arachidic**	-	0.209
**C20:1**	-	0.608
**C20:2**	-	0.734
**C20:3, n6**	-	0.105
**C20:4, Arachidonic**	-	0.262
**C22, Behenic**	-	0.052
**C22:5, Docosapentaenoic**	-	0.080

## Data Availability

Not applicable.

## Abbreviations

NCD	Non-communicable diseases
DMG	Dimethylglycine
TMG	Trimethylglycine
NADPH	Nicotinamide adenine dinucleotide phosphate-hydrogen
GSH	Reduced glutathione
GSSG	Oxidized glutathione
FAD	Flavin adenine dinucleotide
NAD+	Nicotinamide adenine dinucleotide
NADP+	Nicotinamide adenine dinucleotide phosphate
ATP	Adenosine triphosphate
ADP	Adenosine diphosphate
ROS	Reactive oxygen species
HFD	High-fat diet
STD	Standard diet
CTR	Control
VO_2_	Oxygen consumption
VCO_2_	Carbon dioxide production
RQ	Respiratory quotient
EE	Energy expenditure
ALT	Alanine aminotransferase
AST	Aspartate aminotransferase
TG	Triglycerides
IR-HOMA	Insulin resistance-homoeostatic model assessment
TNF-α	Tumor necrosis factor-α
IL-1-β	Interleukin 1-β
IL-6	Interleukin-6
LPS	Lipopolysaccharide
MCP-1	Monocyte chemoattractant protein-1
CPT	Carnitine palmitoyl transferase
AMPK	Adenosine monophosphate-activated protein kinase
p-AMPK	Phosphorylated adenosine monophosphate-activated protein kinase
H_2_O_2_	Hydrogen peroxide
SOD	Superoxide dismutase
NQO1	NADPH quinone dehydrogenase
GST	Glutathione transferase
MDA	Malondialdehyde
PC	Protein carbonyls
FFAs	Free fatty acids
EDTA	Ethylenediaminetetraacetic acid
PBS	Phosphate-buffered saline
TBAR	Thiobarbituric acid
DTNB	Dithionitrobenzoic acid
Nrf2	NF-E2-related factor 2
